# Epithelial cells in bone marrow of oesophageal cancer patients: a significant prognostic factor in multivariate analysis

**DOI:** 10.1054/bjoc.2000.1199

**Published:** 2000-06-02

**Authors:** S Thorban, R Rosenberg, R Busch, R J Roder

**Affiliations:** Department of Surgery, Institute of Medical Statistics and Epidemology, Technische Universität München, Ismaningerstrasse 21, München, D-81675, Germany

**Keywords:** epithelial cells in bone marrow, oesophageal cancer, multivariate analysis, prognosis

## Abstract

The detection of epithelial cells in bone marrow, blood or lymph nodes indicates a disseminatory potential of solid tumours. 225 patients with squamous cell carcinoma of the oesophagus were prospectively studied. Prior to any therapy, cytokeratin-positive (CK) cells in bone marrow were immunocytochemically detected in 75 patients with the monoclonal anti-epithelial-cell antibody A45-B/B3 and correlated with established histopathologic and patient-specific prognosis factors. The prognosis factors were assessed by multivariate analysis. Twenty-nine of 75 (38.7%) patients with oesophageal cancer showed CK-positive cells in bone marrow. The analyses of the mean and median overall survival time showed a significant difference between patients with and without epithelial cells in bone marrow (*P*< 0.001). Multivariate analysis in the total patient population and in patients with curative resection of the primary tumour confirmed the curative resection rate and the bone marrow status as the strongest independent prognostic factors, besides the T-category. The detection of epithelial cells in bone marrow of oesophageal cancer patients is a substantial prognostic factor proved by multivariate analysis and is helpful for exact preoperative staging, as well as monitoring of neoadjuvant therapy. © 2000 Cancer Research Campaign


British Journal of Cancer (2000) **83**(1), 35–39
© 2000 Cancer Research Campaign
doi: 10.1054/ bjoc.2000.1199, available online at http://www.idealibrary.com on IDEAL®

### Epithelial cells in bone marrow of oesophageal cancer patients: a significant prognostic factor in multivariate analysis

**S Thorban,[1] R Rosenberg,[1] R Busch[2] and RJ Roder[1]**

[1]Department of Surgery; [2]Institute of Medical Statistics and Epidemology, Technische Universität München, Ismaningerstrasse 21, D-81675 München, Germany

**Summary** The detection of epithelial cells in bone marrow, blood or lymph nodes indicates a disseminatory potential of solid tumours. 225 patients with squamous cell carcinoma of the oesophagus were prospectively studied. Prior to any therapy, cytokeratin-positive (CK) cells in bone marrow were immunocytochemically detected in 75 patients with the monoclonal anti-epithelial-cell antibody A45-B/B3 and correlated with established histopathologic and patient-specific prognosis factors. The prognosis factors were assessed by multivariate analysis. Twenty-nine of 75 (38.7%) patients with oesophageal cancer showed CK-positive cells in bone marrow. The analyses of the mean and median overall survival time showed a significant difference between patients with and without epithelial cells in bone marrow ($P < 0.001$). Multivariate analysis in the total patient population and in patients with curative resection of the primary tumour confirmed the curative resection rate and the bone marrow status as the strongest independent prognostic factors, besides the T-category. The detection of epithelial cells in bone marrow of oesophageal cancer patients is a substantial prognostic factor proved by multivariate analysis and is helpful for exact preoperative staging, as well as monitoring of neoadjuvant therapy. © 2000 Cancer Research Campaign

**Keywords:** epithelial cells in bone marrow; oesophageal cancer; multivariate analysis; prognosis

In recent years the combination of chemo and/or radiation therapy before surgery has been investigated to prolong survival in patients with squamous cell carcinoma of the oesophagus, but the value of neoadjuvant therapy is currently under debate. In addition to improved surgical resection of primary tumours with adequate lymphadenectomy, preoperative radio/chemotherapy of advanced tumours was another assumption to be locally free of tumour. The lymphadenectomy was especially beneficial in patients with early lymph node metastasis and in subgroups of patients with known lymph node metastasis. With the help of neoadjuvant radio/chemotherapy, the prolongation of entire and disease-free survival could be achieved through preoperative reduction of the primary tumour (Ajani, 1996; Siewert and Roder, 1992; Walsh et al, 1996). Besides the possibility of complete tumour resection, the neoadjuvant therapy could also minimize intraoperative spreading of tumour cells and systemically treat potential existing micrometastases (Herskovic et al, 1992; Siewert et al, 1996).

In spite of the optimized therapy techniques, tumour recurrence occurs in nearly half of all patients with epithelial tumour of the upper gastrointestinal tract within 5 years after initial surgery (Bolton et al, 1996; Law et al, 1996). One reason may be that previously available diagnostic techniques could not detect the dissemination of micrometastatic cells in bone marrow, blood or lymph nodes at the time of surgery. Recently, more sensitive immunocytochemical and molecular methods were developed to detect epithelial cells disseminated into secondary organs (Funke and Schraut, 1998; Johnson et al, 1995; Pantel et al, 1993; Riethmüller and Johnson, 1992). Patients with solid tumours in which epithelial cells were found in bone marrow, despite complete resection of the primary tumour, showed tumour recurrence more frequently throughout the course of disease than patients in which cytokeratin (CK)-positive cells were not found (Lindemann et al, 1992; Moss et al, 1991; Pantel et al, 1993). The question remains, at which time-point does systemic metastasization of oesophageal cancer begin? A possible improvement in tumour staging and in identification of patients with risk of development of tumour recurrence is the demonstration of epithelial cells in bone marrow. The purpose of this prospective study was to examine immunocytochemical epithelial cells in bone marrow of patients with squamous cell carcinoma of the oesophagus. Using a multivariate analysis we determine the role of CK-positive cells in bone marrow at the time of first diagnosis in the prognosis of these patients.

*Received 15 November 1999*
*Revised 7 February 2000*
*Accepted 7 February 2000*

*Correspondence to:* S Thorban

### MATERIALS AND METHODS

### Patients and study design

Between August 1992 and December 1997, a total of 225 patients with histologically proven squamous cell carcinoma of the oesophagus were prospectively admitted and operated upon at our institute. From these 225 patients, 75 agreed to participate in the study. Thirty-eight (59.7%) of the 75 patients were initially operated, while 37 (49.3%) patients underwent neoadjuvant radio/chemotherapy prior to surgery. Patient characteristics are shown in Table 1. In these patients, either tumour resection was initially intended or in cases in which the locally advanced tumour extended beyond the organ, neoadjuvant radio/chemotherapy prior to resection of the primary tumour was performed. Besides a

**Table 1** Characteristics of the patients and primary tumour stage (number of patients (%))

| | All patients (*n* = 225) | Study patients (*n* = 75) |
|---|---|---|
| Male | 178 (79.1) | 65 (86.7) |
| Female | 47 (20.9) | 10 (13.3) |
| Age (range) | 59.3 (22–81) | 55.5 (34–72) |
| Therapy | | |
| Tumour resection | 125 (55.6) | 38 (50.7) |
| Preop. RTx/CTx and tumour resection | 100 (44.4) | 37 (49.3) |
| Tumour stage | | |
| Stage I | 47 (20.9) | 18 (24.0) |
| Stage IIa | 61 (27.1) | 18 (24.0) |
| Stage IIb | 22 (9.8) | 8 (10.7) |
| Stage III | 64 (28.4) | 29 (38.7) |
| Stage IV | 31 (13.8) | 2 (2.7) |
| R-category | | |
| RO-resection | 169 (75.1) | 59 (78.7) |
| R1/2-resection | 56 (24.9) | 16 (21.3) |

RTx/CTx = preoperative radio-/chemotherapy; Ro-resection = curative resection without microscopic/macroscopic residual tumour; R1/2-resection = microscopic and macroscopic residual tumour thorough physical examination to exclude lymph node metastases, the clarification of functional operability was essential for the selection of treatment. Therefore, for each patient a preoperative risk score was assigned in order to objectively determine pulmonary, cardiac, liver and kidney function, as well as cooperativeness, general status (Karnofsky Index > 70%), and bone marrow function. In order to obtain exact preoperative tumour staging, endoscopy and endosonography were under taken for histological biopsies and determination of T-category of the tumour. Furthermore, depending on the condition of the primary tumour, computerized tomography of the neck, chest, or abdomen was performed. The examination was completed with upper abdomen sonography and skeletal scintigraphy to rule out remote metastases (Dittler and Siewert, 1993; Fink et al, 1995). Exclusion criteria for surgery or neoadjuvant therapy included Karnofsky index > 70%, life expectancy less than 3 months, tumour bleeding, secondary tumours, remote metastases, liver cirrhosis, manifest alcohol abuse, peritoneal carcinosis or other remote metastases. The selection of therapy was performed dependently of the resectability of the primary tumour and the overall status of the patient. Surgery-only option was always indicated in cases in which the tumour appears to be completely respectable and it was allowed by the overall status of the patient. In patients with locally advanced tumours and questionable curative resection possibility, the multi-modality concept with preoperative radio/chemotherapy was employed. For the documentation of comparability and representativeness of the examined patients, all patients with surgery and all patients with neoadjuvant therapy from the study were compared to the total patient population. Age, gender, primary tumour status, and RO-resection quota using the Mann-Whitney test were comparable between the two groups. Total survival time was also not significantly different in the operated patients and the neoadjuvant pretreated patients compared to total patients.

### Bone marrow aspiration and immunocytochemistry

After approval of the study by our ethics committee and oral as well written informed consent from the patients, a standardized bone marrow puncture was performed in all patients prior to the start of therapy. The punctures at the iliac spine (anterior superior or posterior superior) were performed either under local anaesthesia or during general anaesthesia at the beginning of the surgery. 2–5 ml bone marrow was obtained and all specimens from a given individual were pooled before further processing. Mononuclear cells (MNCs) were isolated by density-gradient centrifugation by Ficoll-Hypaque (Pharmacia Freiburg, Germany) at 400 *g* for 30 min at room temperature and then deposited onto glass slides by cytocentrifugation at 150 *g* for 8 min, also at room temperature. The number of MNCs spun onto each slide was $2 \times 10^6$. The cytocentrifuge preparations were fixed with acetone for 10 min at room temperature, airdried, and then preincubated with antibody-free human serum for 25 min to block non-specific antibody binding. Epithelial cells were identified using the mouse antocytokeratin monoclonal antibody (MAb) A-45-B/B3, which detects a common epitope present on a variety of cytokeratin components, including cytokeratins 8,18 and 19. The MAb was applied at optimum concentration, ranging from 2–5 $\mu g\ ml^{-1}$, for 45 min at room temperature. For visualization of antibody binding, the sensitive alkaline phosphatase anti-alkaline phosphatase technique (APAAP) was employed (Lindemann et al, 1992; Pantel et al, 1993). Alkaline phosphatase activity was monitored by use of the Neufuchsin stain, and endogenic formation of phosphatase activity was blocked by preincubation with levamisole. The relative proportion of positive cells was recorded quantitatively, differentiating between single cells and cell clusters. For each patient, five slides containing a total of $2 \times 10^6$ MNCs were evaluated by two independent observers in a double-blinded fashion. The specifity of the MAbs as epithelial marker proteins in the bone marrow of patients with solid tumours could additionally be confirmed by examination in 35 disease-free patients of the control group. This control patient group did not differ significantly from the total patient population with respect to gender and age. False-positive immunocytochemical colour reaction with the above-mentioned mAb was not found in any of these patients.

### Surgical and neoadjuvant therapy

All patients with primary resectable squamous cell carcinoma of the oesophagus underwent a one-time transthoracic en-bloc oesophagectomy. After a right thoracotomy, the resection of the oesophagus together with the mediastinal lymph nodes was performed. The reconstruction of the oesophagus was performed through the abdominal and left cervical access either by an upward displacement of the stomach or, where the stomach could not be used due to preoperative or accompanying disease, it was performed by a colon interposition. Patients with locally advanced tumours without remote metastases (stage III, T3,N1,MO) underwent neoadjuvant therapy and then oesophagectomy in two sessions. Four weeks after the end of the radio/chemotherapy through transthoracic surgery the oesophagectomy with lymphadenectomy was performed. Then, 3–4 weeks later, reconstruction was performed in the anterior mediastinum. The preoperative radio/chemotherapy consisted of one cycle of fluorouracil (300 mg $m^{-2}$, given as continuous infusion during radiation therapy) and 3000 cGy (Rad) radiation dose over 3 weeks in 15 sessions Of 200 cGy per day, 5 days per week (Anderson and Lad, 1982; Brockmeyer and Crowley, 1982; Law et al, 1996).

 *© 2000 Cancer Research Campaign*

**Table 2** Patient-specific and histopathologic prognosis factors in multivariate analysis

| |
|---|
| pT category |
| pN category |
| M category |
| R category |
| Grading of the primary tumour (G) |
| Age and gender distribution |
| Overall survival time |
| Tumour stage (according to the UICC-classification 1997) |

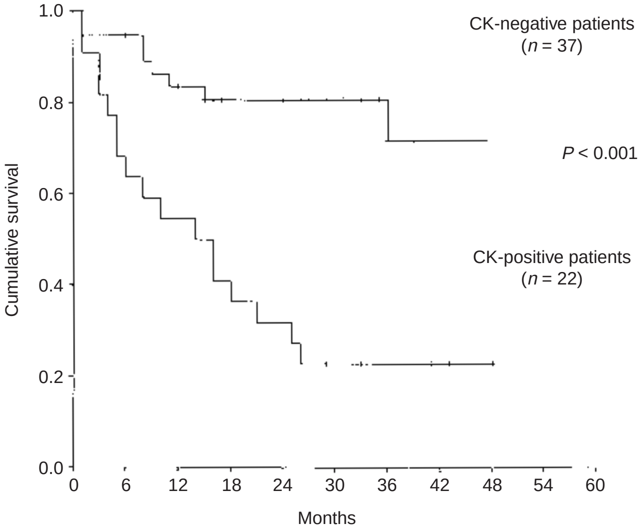

**Figure 1** Overall survival for 59 patients with completely resected (RO) oesophageal cancer according to the presence or absence of CK-positive cells in their bone marrow.

### Data evaluation

The immunocytochemical determined count of epithelial cells in bone marrow of all patients were correlated with established patient-specific and histopathologic prognosis factors (Table 2). Tumour follow-up examinations in the first postoperative year at 3, 6, and 12 months, then every year, were performed by our oncology outpatient unit or by the family physician. In addition to evaluation of clinical symptoms including loss of weight or changes in general status. these examinations consisted of ultrasound examination, as well as X-ray examination, computer-tomography of the chest and abdominal wall and bone scanning for early detection of local recurrence or remote metastases. If there was evidence for tumour recurrence, histological verification was attempted.

The documentation of the patient data was performed according to a standardized study protocol, which was planned and designed under the statistical advice of the Institute for Medical Statistics and Epidemology of the Technischen Universität München. Statistical analysis was performed using a BMDP software package (BMDP Statistical Software Inc., Los Angeles, USA). The survival curve with a 95% confidence interval was calculated from the Kaplan-Meler and Brookmeyer method (Brockmeyer and Crowley, 1982; Kaplan and Meier, 1958). Differences between each patient group were determined using the Log rank test.

**Table 3** Multivariate analysis of prognostic factors in oesophageal cancer patients ($n = 75$)

| Prognostic factor | *P* | Relative risk |
|---|---|---|
| RO category | < 0.000001 | 0.25 |
| Bone marrow state | < 0.0019 | 0.15 |
| T category | < 0.0013 | 0.16 |

The significance was determined using the Mann-Whitney and Chi-Square test, in which $P < 0.05$ was considered as statistically significant. All statistical calculations were performed for the total patient population of the study, and also for the subgroup of patients with curative (RO) resection. For the primary tumour staging and classification of the TNM-category the UICC-classification from 1997 was used (Wittekind and Wagner, 1997).

### RESULTS

### Incidence and frequency of epithelial cells

Altogether, 29 of 75 (38.7%) patients with squamous cell carcinoma of the oesophagus showed CK-positive cells in bone marrow. In 21 (72.4%) of these positive bone marrow samples, less than 10 cells per $2 \times 10^6$ examined MNCs were found. The majority of the patients had few epithelial cells, while clusters of CK-positive cells were found in only 8 (27.6%) of the patients. Significant differences with regard to recurrent rate and total survival time between patients with few epithelial cells, compared to patients with cell clusters in bone marrow, were not found.

### Survival and tumour recurrence

Within the median follow-up period of 35.7 months (3–61 months), the analyses of the mean and median overall survival time showed a significant difference between patients with and without detection of CK-positive bone marrow ($P < 0.001$, Figure 1). Of the 59 patients with RO-resection, 16 (72.7%) of the CK-positive patients and only four (10.8%) of the CK-negative patients developed tumour recurrence during the follow-up period. In eight CK-positive patients, tumour recurrence was found with remote metastases (lung two, liver two, skeletal one, peritoneal one). In another eight patients local recurrence of tumour was observed. Regarding CK-negative patients, one remote metastases and three cases of local tumour recurrence were detected. With respect to age, gender, and distribution of tumour staging, no significant difference between the CK-positive and CK-negative patient subgroups was found. The total number of patients for subgroup analysis was small, but the criteria to draw any firm conclusions were fulfilled by statistical calculation.

### Multivariate analysis

In order to determine independent prognostic factors, a multivariate analysis according to the Cox regression model was performed in the total patient population and in patients with curative (RO) resection. The analysis showed that curative resection of the primary tumour (RO-status), the bone marrow status with detection or lack or CK-positive cells, and the depth of infiltration of primary tumour (T-category) were independent prognostic

*© 2000 Cancer Research Campaign*

factors (Table 3). The bone marrow status and the depth of primary tumour (T-category) were also relevant prognostic factors in the multivariate analysis of exclusively curatively resected patients, after exclusion of RO-status as a prognostic factor.

### DISCUSSION

In various clinical studies, the sensitivity and prognostic relevance of isolated, disseminated epithelial cells in bone marrow of patients with a solid tumour has been documented (Diel and Kaufmann, 1996; Heiss et al, 1995; Johnson et al, 1995; Lindemann et al, 1992; Mass et al 1991; Pantel et al, 1993; Thorban et al, 1996). But the value of epithelial cells in bone marrow as an independent prognostic factor has been critically assessed in a recent meta-analysis (Funke and Schraut, 1998). An average prevalence of approximately 35% of CK-positive cells in bone marrow was found for the various types of carcinoma investigated. Fourteen of the 20 studies analyzed showed, using univariate analysis, a positive correlation between these cells and a reduced tumour recurrence-free survival time. However, only five out of 11 studies confirmed a positive finding in bone marrow as an independent prognostic factor for a disease-free period. The total survival time as an independent prognostic factor is shown in five out of 12 studies using univariate analysis, and in only two studies in the multivariate analysis. The present study is the first report of the detection and the evaluation of prognostic value of epithelial cells in bone marrow of patients with squamous cell carcinoma of the oesophagus. Our analysis showed that the detection of epithelial cells in bone marrow of patients with oesophagus carcinoma has significant prognostic importance. The poor prognosis of CK-positive in comparison to CK-negative patients was confirmed by the significantly decreased total survival time ($P < 0.001$). Furthermore, in the multivariate analysis of all prognostic factors, the detection of CK-positive cells represented an independent prognostic factor in addition to the T- and R-category.

The destiny of these epithelial cells in bone marrow can have various courses. While some cells can become precursors for skeletal metastases, others leave the bone marrow and can settle and develope metastases in various other organs. This hypothesis may explain the very low number of skeletal metastases in CK-positive patients in the present study, which is in concordance to other reported studies (Anderson and Lad, 1982; Mandard et al, 1981). Epithelial cells in bone marrow appear to indicate the transition of the state of tumour disease, which on one hand could show a general spreading of the primary tumour, but not necessarily a metastasization. In many studies bone marrow is used as the indicator organ because it is easily accessible and is normally devoided of epithelial cells. So far, analysis of two aspirates from both sites of the iliac crest seems to be sufficient to detect about 90% of patients with epithelial cells in bone marrow. Recent results showed that detection rates based on iliac crest marrow are underestimated. O'Sullivan et al (1999) found in bone marrow of the rib in 88% of his patients epithelial cells.

Another important point may be the different results from immunohistochemistry if polymerase chain reaction (PCR) methods were employed. Theoretically, the PCR method should be the most sensitive method to detect epithelial cells in bone marrow or lymph nodes. Until now it was difficult to establish a respective approach for screening such patients, because of the tumour cell heterogeneity. Although such assays are extremely sensitive, their specifity is limited by the low-level ectopic expression of cytokeratin mRNA in nonepithelial cells. While such contamination can be discriminated by the cytologic approach used here, it cannot be distinguished by RT-PCR.

**Table 4** Incidence of CK-positive cells in bone marrow and micrometastasis of lymph nodes in oesophageal cancer patients (*n* = 24)

| Bone marrow state | Micrometastasis of lymph nodes | *n* (%) |
|---|---|---|
| Positive | Positive | 3 (12.5) |
| Positive | Negative | 4 (16.5) |
| Negative | Positive | 5 (20.8) |
| Negative | Negative | 12 (50.0) |

Correspondence of bone marrow state and lymp node state in 15 (62.5%) patients

As previously mentioned, detection of micrometastases is not only possible in bone marrow but also in lymph nodes, despite negative conventional histopathologicic findings. A previous study at our institute with 69 oesophageal cancer patients with complete resection of the primary tumour, it was shown that with regard to survival time the detection of micrometastases in cases of 'histologically tumour-free' lymph nodes prognostically corresponded to a lymph node metastasization (Natsugoe et al, 1998). In 24 of the 69 (34.8%) patients, epithelial cells in bone marrow were screened during the same observation period. The correlation of bone marrow status with micrometastases in lymph nodes is presented in Table 4. A correspondence concerning micrometastases between bone marrow state and lymph node state was found in 15 (62.5%) patients. Izbickl et al (1997) support the prognostic importance of micrometastases in lymph nodes of oesophageal cancer patients with regard to the decreased overall survival and recurrence-free survival time. In all of the 25 patients of this study with CK-positive bone marrow status, a positive lymph node involvement was also found. Furthermore, in 40% of the patients with CK-negative bone marrow, an immunocytologically positive lymph node involvement was detected. On the contrary, Glickman et al, (1999) reported, in 78 oesophageal carcinoma patients, no correlation between the detection of occult lymph node metastases and any of the clinical or histopathologic prognostic factors. Therefore, he proposed no further immunocytologic examination of lymph nodes in order to improve tumour staging.

The occurrence of CK-positive cells in bone marrow indicates precise tumour staging with haematogenous dissemination of malignant cells leading to an increased risk of metastatic relapse. Thus far, the influence of neoadjuvant therapy on the appearance and change of CK-positive cells in bone marrow of oesophageal cancer patients has not been thoroughly investigated. In the course of the disease all patients died after 2–5 years due to remote metastases inspite of their response to neoadjuvant therapy Herskovic et al, 1992; Wolfe et al, 1993. That fact raises the question if inadequate chemotherapy was given to manage metastatic or even micrometastatic disease. Previous analyses of micrometastatic tumour cells in the bone marrow of patients with gastrointestinal carcinomas indicated that the majority of these cells are in a nonproliferative state, which explains the relative ineffectiveness of a systemic chemotherapy (Pantel, 1993). Furthermore, it is unclear whether CK-positive cells surviving in bone marrow after RTx/CTx have the same metastatic potential as the cells present before neoadjuvant therapy. A major objective of future investigations will be the precise characterization of the phenotype of these

© 2000 Cancer Research Campaign epithelial cells, e.g. using fluorescence in situ hybridization, in order to improve the understanding of the oncogene potential of these cells. The multivariate analysis, first carried out in our population of oesophageal cancer patients, underlines that the detection of these cells represents an independent prognostic factor. Further investigations regarding the effect of a combined neoadjuvant and antibody therapy with resection of the primary tumour on these cells in bone marrow are necessary. As suggested by this study, immunocytological detection of CK-positive cells might be helpful for precise preoperative tumour staging and monitoring of tumour response after neoadjuvant therapy in squamous cell oesophageal cancer patients.

### ACKNOWLEDGEMENTS

We dedicate this manuscript to our surgical and scientific teacher Prof J Rüdiger Siewert for his 60th birthday. This work was supported by grants from the Wilhelm-Sander-Stiftung, Neuburg/Donau and the Friedrich-Baur-Stiftung, Germany.

### REFERENCES

Ajani JA (1996) Therapy of carcinoma of the oesophagus: either attempt it not or succeed. *Eur J Cancer* **31A**: 790–793

Anderson LL and Lad TE (1982) Autopsy findings in squamous cell carcinoma of the oesophagus. *Cancer* **50**: 1587–1590

Bolton JS, Ochsner JL and Abdoh AA (1994) Surgical management of oesophageal cancer. A decade of change. *Ann Surg* **219**: 475–480

Brookmeyer R and Crowley J (1982) A confidence interval for the median survival time. *Biometrics* **38**: 29–41

Diel IJ and Kaufmann M (1996) Micrometastatic breast cancer cells in bone marrow at primary surgery: prognostic value in comparison with nodal status. *J Natl Cancer Inst* **88**: 1652–1656

Dittler HJ and Siewert JR (1993) Role of endoskopic ultrasonography in oesophageal cancer. *Endoscopy* **15**: 156–161

Fink U, Stein HJ, Wilke HJ and Siewert JR (1995) Multimodal treatment for squamous cell oesophageal cancer. *World J Surg* **19**: 198–204

Funke I and Schraut W (1998) Meta-analyses of studies on bone marrow micrometastases: an independent prognostic impact remains to be substantiated. *J Clin Oncol* **16**: 557–566

Glickman JN, Torres C, Wang HH, Turner JR, Shahsafael A, Richards WG, Sugarbaker DJ and Odze RD (1999) The prognostic significance of lymph node micrometastasis in patients with oesophageal carcinoma. *Cancer* **15**: 769–778

Heiss MM, Allgayer H, Gruetzner KU, Funke I, Babic R, Jauch KW and Schlldberg FW (1995) Individual development and uPa- receptor expression of disseminated tumor cells in bone marrow: A reference to early systemic disease in solid cancer. *Nat Med* **1**: 1035–1039

Herskovic A, Martz K and Al-Sarraf M (1992) Combined chemotherapy and radiotherapy compared with radiotherapy alone in patients with cancer of the oesophagus. *N Engl J Med* **326**: 1593–1598

Izbicki JR, Hosch SB, Pichimeier U, Rehders A, Busch C, Niendorf A, Passlick B, Broelsch CE and Pantel K (1997) Prognostic value of immunohistochemically identifiable tumor cells in lymph nodes of patients with completely resected oesophageal cancer. *N Engl J Med* **337**: 1188–1194

Johnson PWM, Burchill SA and Selby PJ (1995) The molecular detection of circulating tumour cells. *Br J Cancer* **72**: 268–273

Kaplan EL and Meyer P (1958) Nonparametric estimation from incomplete observations. *Journal of the American Statistical Association* **53**: 457–481

Law SYK, Fok M and Wong J (1996) Pattern of recurrence after oesophageal resection for cancer: clinical implications. *Br J Surg* **83**: 107–111

Lindemann F, Schlimok G, Dirschedl P, Witte J and Riethmüller G (1992) Prognostic significance of micrometastatic tumour cells in bone marrow of colorectal cancer patients. *Lancet* **340**: 685–689

Mandard AM, Chasle J, Marnay J, Villedleu B, Blanco C and Roussel A (1981) Autopsy findings in 111 cases of oesophageal cancer. *Cancer* **48**: 329–335

Moss TJ, Reynolds CP, Sather HN, Romansky SG, Hammond GD and Seeger RC (1991) Prognostic value of imunocytological detection of bone marrow metastases in neuroblastoma. *N Engl J Med* **324**: 219–226

Natsugoe S, Mueller J, Stein HJ, Feith M, Höfler H and Siewert JR (1998) Micrometastasis and tumor cell microinvolvement of lymph nodes from oesophageal squamous cel carcinoma. *Cancer* **83**: 858–866

Pantel K, Schllmok G, Braun S, Kutter D, Schaller G, Funke I, Izbicki JR and Riethmüller G (1993) Differential expression of proliferation-associated molecules in individual metastatic carcinoma cells. *J Natl Cancer Inst* **85**: 1419–1425

O'Sullivan GC, Sheehan D, Clarke A, Stuart R, Kelly J, Kiely MD, Walsh T, Collins JK and Shanahan F (1999) Micrometastases in oesophagogastric cancer: high detection rate in resected rib segments. *Gastroenterology* **116**: 543–548

Riethmüller G and Johnson JP (1992) Monoclonal antibodies in the detection and therapy of micrometastatic cancers. *Curr Opin Immunol* **3**: 647–655

Siewert JR and Roder JD (1992) Lymphadenectomy in oesophageal cancer surgery. *Diseases of the Oesophagus* **5**: 91–97

Siewert JR, Stein HJ and Fink U (1996) Multimodality therapy for oesophageal cancer. *The Oncologist* **1**: 210–218

Thorban S, Roder JD, Pantel K and Siewert JR (1996) Immunocytochemical detection of isolated epithelial tumor cells in bone marrow of patients with pancreatic carcinoma. *Am J Surg* **172**: 297–298

Waish TN, Noonan N, Hollywood D, Kelly A, Keeling N and Henessy TPJ (1996) A comparison of multimodal treatment and surgery for oesophageal adenocarcinoma. *N Engl J Med* **335**: 462–467

Wittekind CH and Wagner G (1997) UICC: TNM-Klassifikation maligner Tumoren, 5. Auflage, Springer-Verlag: Berlin, Heidelberg: New York

Wolfe WG, Vaughn AL, Seigler HF, Hathorn JW, Leopold KA and Duhayionsod FG (1993) Survival of patients with carcinoma of the oesophagus treated with combined modality therapy. *J Thorac Cardvasc Surg* **105**: 749–755

*© 2000 Cancer Research Campaign*
